# COVID-19 adaptive evolution during the pandemic – Implications of new SARS-CoV-2 variants on public health policies

**DOI:** 10.1080/21505594.2021.1960109

**Published:** 2021-07-29

**Authors:** Cock van Oosterhout, Jessica F. Stephenson, Bart Weimer, Hinh Ly, Neil Hall, Kevin M. Tyler

**Affiliations:** School of Environmental Sciences, Norwich Research Park, University of East Anglia, UKc.van-oosterhout@uea.ac.uk; Department of Biological Sciences, University of Pittsburgh, Pittsburgh, PA, USA; School of Veterinary Medicine, Population Health and Reproduction, 100K Pathogen Genome Project, University of California Davis, Davis, CA, USA; Department of Veterinary & Biomedical Sciences, University of Minnesota Twin Cities, St. Paul, MN, USA; Earlham Institute, Norwich Research Park, Norwich, UK; Center of Excellence for Bionanoscience Research, King Abdul Aziz University, Jeddah, Saudi Arabia; Norwich Medical School, University of East Anglia, Norwich, UKk.tyler@uea.ac.uk

The development and deployment of effective vaccines against SARS-CoV-2 infection have boosted public confidence and heralded a change in public health policies. The wearing of facemasks and social distancing has become increasingly relaxed in some countries, and large gatherings are again permitted. Here, we provide a biological argument against relaxing the COVID-19 restrictions during the exponential growth phase of infections whilst a significant proportion of the population remains unvaccinated (the so-called “British Experiment”). Using coevolutionary and epidemiological theory, we explain the likely long-term consequences of this policy, and we argue for the benefits of “vaccination passports” and childhood vaccination.

Although vaccines have weakened the link between COVID-19 infection and mortality in some countries, using this as an argument to justify a broad change in policy for countries experiencing an exponential increase in infection numbers is flawed on two counts. First, most of the world’s population remains unvaccinated, and even in countries with efficient vaccination programmes, a significant proportion of the society remains unprotected. Children, the clinically extremely vulnerable, such as patients on immunosuppressants, and those who choose not to be vaccinated (or are unable to receive a vaccine) are all placed at an increased risk when relaxing COVID-19 restrictions. Initial analysis of UK wide data on the “Clinical Extremely Vulnerable” group of immunosuppressed transplant patients both shows the significant advantages of vaccination in this group, and simultaneously demonstrates that the risk remains at a level substantially above that of the general population [1, personal communication]. Consequently, without “vaccination passports” being enforced in crowded public places, vulnerable people are unable to manage their personal risks when traveling or socializing in a society with little or no COVID-19 restrictions.

Second, relaxing restrictions boosts transmission and allows the virus population to expand, which enhances its adaptive evolutionary potential and increases the risk of vaccine-resistant strains emerging through antigenic drift [2]. A fundamental theory of biology is that the rate of adaptive evolution is a function of the (additive) genetic variation present in a population or gene pool. This is known as Fisher’s fundamental theorem [3], and it implies that the adaptive evolutionary potential (present in an assortment of new variants) tends to increase with population size. In the case of a directly transmitted pathogen, such as SARS-CoV-2, this evolutionary potential scales positively with the number of infected hosts. Thus, by minimizing this number, we reduce not only the current COVID-19 related mortality rate but also future COVID-19 related deaths by restricting the rate with which new SARS-CoV-2 variants arise.

As with any other host species, humans are engaged in a coevolutionary arms race with emerging infectious diseases, including SARS-CoV-2 [4]. However, unlike any natural arms race, humans fight this pandemic battle with an unusual type of armory; our scientific and medical advances, as well as our behavioral and societal changes. In the past 18 months, economies, education, and mental well-being have suffered tremendously due to the restrictions imposed in an attempt to stem the spread of the pandemic. These restrictions have reduced mortality rates and bought us time to develop and employ effective vaccines. Nevertheless, the virus has continued to evolve increased transmissibility, and even in some countries with the most advanced vaccination programs, the reproduction number *R_t_* of the virus (i.e. the mean number of new infections arising from each infected individual at time t) is similar to the *R*_0_ of first wave of infection. Despite the success of vaccines and public health policies globally, the number of infected people continues to fluctuate (Figure 1(a)), which is typical of coevolution. Like in so many other Red Queen arms races, there are no winners – coevolution is a zero-sum game [5]. Importantly, we need to realize that it is unlikely we will get ahead in this arms race unless we can significantly reduce the population size of the virus. We therefore need to contain the adaptive evolutionary potential of this virus, and this can only be accomplished by a worldwide uptake of the vaccine. Until that time, other social mitigation strategies will need to continue, despite the costs these entail.

**Figure 1.**
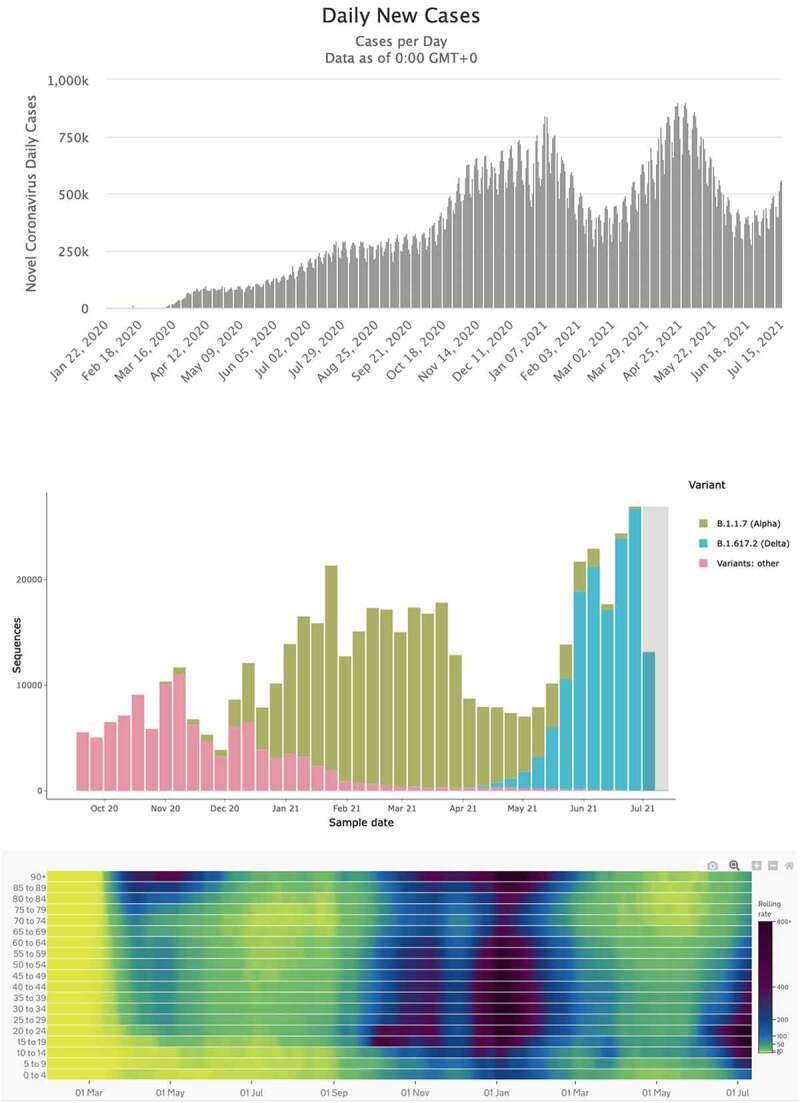
(a) The global number of daily cases of COVID-19 fluctuates, similar as other co-evolutionary arms races. In spite of public health campaigns and vaccine rollout, the number is not declining consistent with coevolution of the virus (From https://www.worldometers.info/coronavirus/ 17 July 2021). (b) Histogram from COG-UK shows the variant sequences obtained in the UK since October 2020. The emergence of a new variant (delta variant) displaces its ancestral variant (alpha variant) – a pattern typical for Red Queens arms race in host-parasite co-evolution. From http://sars2.cvr.gla.ac.uk/cog-uk/ 17 July 2021 (c) Heatmap of COVID-19 Cases in England between March 2020 to July 2021 broken down by age group. School age children remained largely unaffected in the first wave of the infection, but not the case in the second wave (alpha variant) and in the third wave (driven by the delta variant), high school age children and young adults are the most likely groups to be infected. From https://coronavirus.data.gov.uk/details/cases?areaType=nation&areaName=England 17 July 2021

Successive SARS-CoV-2 variants that have displaced one another since the outbreak (Figure 1(b)), have managed to do so based on relatively small sets of substitutions in their genetic code. More specifically, variants of concern such as B.1.1.7, B.1.351 and P.1 are characterized by the accumulation of mutations in the spike glycoprotein (S), as well as in other genes [2]. Each successive dominant strain displaced its ancestral variant by the fact of having a higher transmissibility [6]. This process is known as antigenic drift, and it has largely offset the gains we have made with vaccination programs. Indeed, our battle with the virus it typical of the zero-sum game of “tit-for-tat” coevolution in a Red Queen’s arm race. Empirical evidence, modeling data, and experiments on mouse-models furthermore suggest that increased transmissibility attributable to increased viremia may also lead to an increased virulence [7, 8, 9, 10], although this is not always the case [11]. Slowing down the rate of virus evolution requires us to act fast and decisively, reducing the number of infected people through the use of vaccines, in combination with other public health policies. On the human side, the arms race is fought with vaccines and behavioral change, but the principles are the same as genetic adaptations. Our recently acquired behavioral changes offer defense in the arms race against this pathogen, and those changes are adaptive and should be reinforced by public health policies. Relaxing these measures now would be letting our guard down in the middle of the battle, and it would place us at a significant disadvantage in the arms race with the virus.

## Child vaccination

The observed shift in the ages most affected by subsequent variants of COVID-19 (Figure 1(c)) may be as a result of the adaptive evolution of SARS-CoV-2 to its human host. The fixation of subsequent variants of increased virulence, can be understood from the “virulence-transmission trade off” hypothesis [12,13]. This hypothesis states that pathogen replication poses an unavoidable cost to its host, i.e., the pathogen’s “virulence”. Unvaccinated children represent a large reservoir that is susceptible to infection but tolerant to the disease. Within this group, SARS-CoV-2 continues to evolve. As such, this unvaccinated part of the population puts more vulnerable groups at increased risk because of 1) elevated transmission rates and 2) continued evolution of the virus. The low level of morbidity associated with COVID-19 in most children argues against vaccinating this age group, as the vaccination of children could be considered unethical if it protects against a disease that carries only a small risk to a child’s health [14]. However, as soon as SARS-CoV-2 variants evolve with significant virulence in this age group, child vaccination may be needed to control antigenic drift. Transmissibility, increased pathology, and viral titers are a linked triad, and hence, the evolution of new strains with higher transmissibility in children may also result in increased disease severity. In other words, this policy risks inadvertently selecting for variants that are better able to infect children, with higher titers and greater pathology in this previously largely refractory age group. Secondly, although vaccine driven virulence evolution remains only a theoretical possibility, the risks of a more virulent strain spreading into an under-vaccinated population could be more severe [15].

## Conclusion

Entrusting public health measures to personal responsibility, the laissez-faire approach that many governments are now taking toward COVID-19 management, risks substantially increasing mortality and morbidity in the wider population. During exponential transmission of virus, we argue for an ongoing, mandatory public health policy that includes social distancing and the compulsory wearing of facemasks in crowded indoor spaces such as shops and on public transport. Epidemiological evidence suggests that we can only safely start to relax these measures when the reproductive number (Rt) of the infection is below one. Our current vaccination programmes alone will not end the pandemic. Reaching long term accommodation with this virus requires immunity of nearly the whole world’s population either by vaccine or infection. Vaccines offer an affordable and highly effective mechanism for delivering immunity without the substantial risks from infection which include acute illness and long COVID. The risk associated with large scale natural infection is not predictable because we cannot know the virulence characteristics and vaccine susceptibility of the new variants that will continue to evolve. With an increasing number of people being infected, the rate of new variant evolution of SARS-CoV-2 accelerates, increasing the likelihood that SARS-CoV-2 variants will acquire higher virulence and vaccine escape potential. Such new variants could rapidly replace existing strains, particularly during the exponential growth phase of the infection. In the long-term, this would place increasing pressure on our societies and economies. To keep ahead in the battle against this virus, we need to take a long-term co-evolutionary perspective. We must not drop our guard in this arms race, as the virus continues to evolve, and we therefore must sustain global public health policies which are proven to reduce the transmission rate of this virus.
